# Cervids as a Promising Pillar of an Integrated Surveillance System for Emerging Infectious Diseases in Hungary: A Pilot Study

**DOI:** 10.3390/ani15131948

**Published:** 2025-07-02

**Authors:** István Lakatos, Péter Malik, Kornélia Bodó, Zsuzsanna Szőke, Farkas Sükösd, Zsófia Lanszki, László Szemethy, Kornélia Kurucz, Krisztián Bányai, Gábor Kemenesi, Brigitta Zana

**Affiliations:** 1 Department of Regional Game Management, Ministry of Agriculture, 1052 Budapest, Hungary; istvan.lakatos@am.gov.hu; 2 Department of Virology, National Food Chain Safety Office VDD, 1143 Budapest, Hungary; malikp@nebih.gov.hu; 3 National Laboratory of Virology, Szentágothai Research Centre, University of Pécs, 7624 Pécs, Hungary; bodo.kornelia@pte.hu (K.B.); lanszki.zsofia@pte.hu (Z.L.); kurucz.kornelia@pte.hu (K.K.); kemenesi.gabor@pte.hu (G.K.); 4 Department of Animal Biotechnology, Institute of Genetics and Biotechnology, Hungarian University of Agriculture and Life Sciences, 2100 Gödöllő, Hungary; ferenczine.szoke.zsuzsanna@uni-mate.hu; 5 Institute of Pathology, University of Szeged, 6720 Szeged, Hungary; sukosd.farkas@gmail.com; 6 Faculty of Sciences, Institute of Biology, University of Pécs, 7624 Pécs, Hungary; szemethy.laszlo@pte.hu; 7 Department of Medical Biology, Medical School, University of Pécs, 7624 Pécs, Hungary; bkrota@hotmail.com; 8 Department of Pharmacology and Toxicology, University of Veterinary Medicine, 1078 Budapest, Hungary

**Keywords:** monitoring, serology, sentinel reservoir, *Flavivirus*, *Orbivirus*, vector-borne pathogens, zoonosis

## Abstract

Wildlife serves as a significant reservoir for various pathogens transmissible to domestic animals and humans. Vector-borne diseases represent an increasing concern in Europe, affecting both animal and human health. This pilot study investigated the circulation of endemic and emerging vector-borne viruses in wild ungulates in Hungary, utilizing a One Health approach. Serum samples were obtained from European fallow deer (*Dama dama*), red deer (*Cervus elaphus*), and roe deer (*Capreolus capreolus*) during routine national game management activities between 2020 and 2023. Samples were analyzed for antibodies against the Bluetongue virus (BTV), West Nile virus (WNV), and Epizootic hemorrhagic disease virus (EHDV) using ELISA and neutralization tests. The results revealed a WNV seroprevalence of 22.3% in fallow deer and 31.8% in red deer, while BTV seroprevalence was 2.5% in fallow deer. All samples were negative for EHDV antibodies. These findings confirm the circulation of WNV and BTV in Hungarian wild ungulates. While the study’s design precludes statistical analysis due to non-random sampling, it demonstrates the potential of integrating wild ungulate serosurveillance into disease monitoring programs, leveraging established wildlife management activities for a cost-effective and complementary approach to One Health surveillance, particularly considering the ongoing spread of EHDV in Europe and the importance of BTV serotype monitoring for effective vaccination strategies.

## Simple Summary

This study shows that wild animals such as deer can serve as indicators for diseases that might later affect farm animals and people. In the frame of routine wildlife management, blood samples from wild deer were collected over several years in Hungary and analyzed for signs of viral infections spread by biting insects, which can cause illness in both animals and humans. The results revealed that many deer had been exposed to a virus similar to West Nile virus, while only a few showed evidence of another virus that can affect livestock. These findings suggest that by monitoring wild deer, authorities will gain a more comprehensive picture of the spread of various diseases, which can contribute to a more effective response to epidemics in the future. This approach offers a practical and affordable addition to current animal health monitoring systems, ultimately helping to protect both public health and the farming community.

## 1. Introduction

The majority of infectious diseases originate from the wildlife interface. Wild animals are important hosts and reservoirs for numerous pathogens of domestic animals and humans [1].

Among infectious diseases, vector-borne pathogens have been particularly prevalent in recent decades, a process that is largely attributed to the increase in blood-sucking arthropods resulting from increasing human activities and environmental changes. Vector-borne pathogens are not only important from a human health perspective but also play a significant role in the diseases of farm animals. Since wild animals have a frequent and diverse connection with pathogen vectors, the investigation of certain animal groups may be an efficient element of an integrated One Health surveillance strategy [2]. Europe has faced the emergence of multiple vector-borne pathogens during the last decades, and current trends are forecasting the growing nature of this trend [3,4].

Among others, the Bluetongue virus (BTV) and the Epizootic hemorrhagic disease virus (EHDV) are arboviruses of outstanding veterinary importance. The two viruses are closely related to each other in the genus *Orbivirus*, and their primary vector species are members of the genus *Culicoides*. Although both viruses can occur in the wild and farmed ungulates, BTV infection causes significant economic damage in livestock, while EHDV causes epidemics mainly in wild ungulates. Even though EHDV infection is generally subclinical in farm animals, recent cases of EHDV epidemics have been reported in farm animals, for example, from the Mediterranean region. Although Central and Northern Europe is BTV-free thanks to the measures taken, the circulation of the virus in the Mediterranean region continues to cause a significant problem [5]. Another important aspect of controlling the spread of BTV and EHDV is the vaccination strategy. BTV- and EHDV-specific vaccines (live attenuated and inactivated) are serotype-dependent, so the most crucial pillar of vaccination is the continuous monitoring of the presence of different serotypes of the viruses in a given area. The continuous outbreaks and emergence of new serotypes, accelerating the development of recombinant vaccines, may provide cross-protection against multiple BTV and EHDV serotypes [6].

In addition to the two viruses of animal health importance mentioned above, the West Nile virus (WNV) is of particular importance in the region and a current example of inter-species transmission with both animal and human health importance. Although birds are the primary reservoir for the WNV (bird–mosquito enzootic cycle) and play a major role in the persistence of the virus in nature, the role of wild ungulates has also emerged as a sentinel animal group [7,8].

In recent decades, the possible role of wild ungulates in maintaining vector-borne pathogens and their vectors has been raised regarding infectious diseases of domestic ruminants and humans. The overpopulation of wild ungulates creates favorable conditions for increasing exposure to infectious diseases and their vectors, creating an optimal environment for the silent maintenance of vector-borne viruses in nature [7,9].

Effective disease surveillance necessitates a “One Health” approach that acknowledges the interconnections between human, animal, and environmental health [2]. While passive surveillance of wild birds provides valuable data regarding WNV activity [10], investigating other animal reservoirs is crucial for a comprehensive understanding of pathogen dissemination. Specifically, wild ungulates may be susceptible to various viruses and potentially serve as indicators of broader disease activity. This pilot study utilizes blood samples collected from wild ungulates during national wildlife management activities to investigate the presence of endemic and emerging viruses in Hungary. The objective is to evaluate the feasibility and efficacy of incorporating wild ungulate serosurveillance into existing disease surveillance programs, in accordance with the One Health approach.

## 2. Materials and Methods

### 2.1. Sample Collection

**Figure 1:**
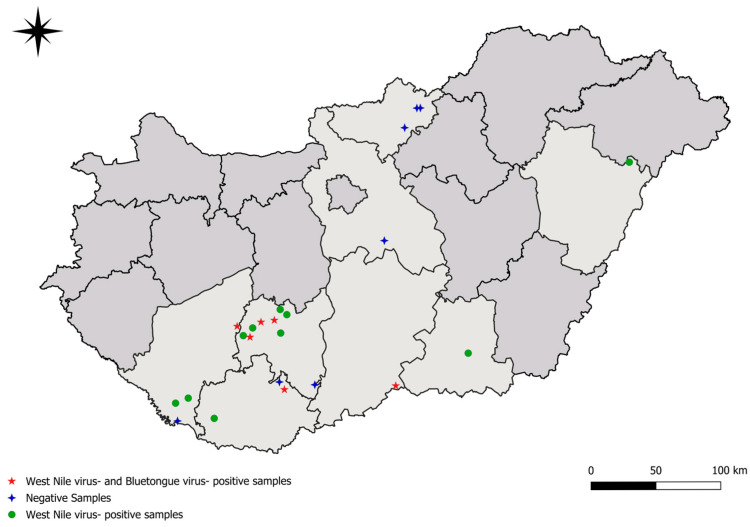
Locations of samples’ origin. Green dots represent the origin of West-Nile virus (WNV)-positive samples. Red stars represent the locations of WNV- and Bluetongue virus (BTV)-positive samples. Blue crosses represent the locations of negative samples for WNV and BTV.

Sampling was conducted during the hunting seasons between 2020 and 2023 in 16 game units in Hungary (Figure 1). Regarding European fallow deer (*Dama dama*) and red deer (*Cervus elaphus*), hind sampling occurred during planned driven hunts, whereas stag hunting was performed individually. Roe deer (*Capreolus capreolus*) hunting was consistently carried out individually. Blood samples from wild ungulates were collected by hunters; whole blood was obtained from the pulmonary artery using a 5 mL syringe. The samples were maintained at 4 °C. Following transportation of the blood samples to the laboratory, the serum was separated by centrifugation for 10 min. Serum samples were stored at −20 °C until experimental use. As per the statement of the Institutional Review Board (NAIK MBK MÁB 004-09/2018), the study is not classified as an animal experiment, as the researchers obtained blood samples from legally harvested European fallow deer, red deer, and roe deer; consequently, the ethical treatment protocols are not applicable. Detailed data of collected samples are available in Supplementary Table S1.

### 2.2. ELISA

All ELISA tests were performed according to the manufacturer’s instructions.

Antibodies against the BT virus were tested with the ELISA kit manufactured by ID Vet, Grabels, France (ID Screen^®^ Bluetongue Competition test). This competitive ELISA kit detects anti-VP7 antibodies in serum or plasma from multiple species. It can be used for the detection of antibodies against all BTV serotypes, at least 5–8 days after natural infection, thanks to the use of a monoclonal antibody against the highly conserved VP7 protein. The testing procedure begins with the dilution of the blood samples and then an incubation period of 45 min at 21 °C. Then, without a washing step, a conjugate is added, and there is another incubation step for 30 min at 21 °C. After a washing step, a substrate solution is added, and after 15 min, the reaction is stopped with the stop solution. The optical density of each well should be measured at 450 nm.

Another competitive ELISA was used for the detection of antibodies against EHDV. The ID Screen^®^ EHDV Competition ELISA (ID.Vet) is capable of detecting the antibodies against the EHDV VP7 protein in sheep, goat, cattle, buffalo, or deer in serum and plasma samples. The test detects all EHDV serotypes, as the VP7 protein is highly conserved, and can give a positive result after 7–15 days post-infection. The first step is the dilution of the blood samples, followed by an incubation period of 45 min at 21 °C. Then, after a washing step, conjugate is added, and there is another incubation step for 30 min at 21 °C. After a washing step, a substrate solution is added, and after 15 min, the reaction is stopped with the stop solution. The optical density of each well should be recorded at 450 nm.

For the detection of anti-flavivirus antibodies, the ID Screen^®^ Flavivirus Competition test (ID.Vet) was used. The kit is designed to detect a wide range of anti-flavivirus antibodies (West Nile virus (WNV), Japanese encephalitis virus (JEV), Tick-borne encephalitis virus (TBEV), Usutu virus (USUV), Zika virus (ZIKAV), and Dengue virus (DENV)) in samples originating from multiple species. The procedure is very similar to the previous methods; it also begins with the dilution of the blood samples, and then there is the first incubation period of 90 min at 21 °C. After a washing step, a conjugate is added, and there is another incubation step for 30 min at 21 °C. After the next washing step, a substrate solution is added, and after 15 min, the reaction is stopped with the stop solution. The optical density of each well should be measured at 450 nm.

### 2.3. Virus Neutralization Assay

Flavivirus-positive samples were further tested by ELISA and anti-WNV neutralization tests to exclude non-WNV-specific positive results due to high cross-reactivity among flaviviruses and the universal flavivirus detection nature of the ELISA kit. Virus neutralization tests (VNTs) were performed at the BSL-4 laboratory of the University of Pécs. Anti-WNV VNTs were performed on Vero-E6 cells with 70% confluency using a 1:10 dilution of the serum samples and 1000 TCID50 of WNV stock. Plates were incubated at 37 °C with 5% CO_2_ for 7 days with continuous monitoring for cytopathic effect. For a more accurate evaluation of the results, after incubation, plates were fixed in methanol and examined by indirect immunofluorescence staining (IF). An indirect IF test was carried out with Anti-dsRNA monoclonal antibody J2 (Nordic-Mubio, Susteren, The Netherlands) at a 1:800 dilution and goat anti-mouse Alexa Fluor 488 (Invitrogen, Waltham, MA, USA) at a 1:1000 dilution. The results were screened under an immunofluorescent microscope. To exclude background fluorescence and autofluorescence, we prepared two negative wells stained with both primer and secondary antibody and two wells stained only with secondary antibody. No cutoff value was determined in this assay.

### 2.4. Detection of BTV by Real-Time RT-PCR

To strengthen the results of ELISA tests and to determine the serotype of BTV circulating among wild ungulates, we performed real-time RT-PCR. For PCR, we used the primers designed by Hofmann et al. (2008) [11] and the Luna^®^ Universal Probe One-Step RT-qPCR Kit (New England Biolabs, Ipswich, MA, USA) according to the manufacturer’s protocol.

## 3. Results

A total of 342 serum samples were tested. Anti-BTV antibodies were detected by ELISA in 8/318 (2.5%) European fallow deer, 0/22 (0%) red deer, and 0/1 (0%) roe deer sera. In the case of WNV, anti-WNV antibodies were detected by ELISA in 94/318 (29.6%) European fallow deer, 9/22 (40.9%) red deer, and 0/1 (0%) in roe deer sera. The anti-WNV antibody-specific virus neutralization test confirmed 22.3% (71/318) and 31.8% (7/22) overall seroprevalence in serum samples from European fallow deer and red deer, respectively. Regarding EHDV, all examined samples were negative by ELISA (Table 1), while we found WNV- and BTV-seropositive animals in all the sampling periods (Table 2). During PCR screening of BTV-positive samples by ELISA, we were unable to detect the viral RNA in any of the samples; therefore, we were unable to determine the circulating serotype in wild ungulates.

## 4. Discussion

In this study, we examined wild ungulate serum samples collected from 2020 to 2023 to investigate the possible circulation of different vector-borne pathogens with human and animal health concerns.

The overall prevalence of WNV-specific antibody detected in this study in European fallow deer (22.3%) and red deer (31.8%) is consistent with the results of several previous studies of serum samples from different artiodactyls and red foxes examined in other European countries [8,12,13]. Additionally, a lower seroprevalence of WNV within wild and domestic artiodactyls has also been found in the literature [14,15,16].

To assess how the findings of this study relate to the West Nile virus (WNV) situation in Hungary, we compared our data (Figure 1, Table 1) with a previous comprehensive study focusing on birds, horses, and humans. Based on this comparison, we can conclude that our seropositive wild ungulate cases show significant geographic overlap with the detected cases in the earlier study [17]. This further supports the hypothesis [18,19] that wild ungulates may play a crucial role as sentinel reservoir hosts in the maintenance of WNV in Hungary as well.

Moreover, the results of the universal flavivirus ELISAs show higher seroprevalence compared to the results of WNV-specific VNTs, suggesting that other flaviviruses are also circulating among wild ungulates, highlighting the importance of including other flaviviruses in the surveillance program.

Regarding BTV-specific antibody detection, our results show much lower seroprevalence among the examined wild ungulates in Hungary than in the case of other European countries [18,19]. Comparing our results to a previous Hungarian study focusing mainly on domestic ruminants, the BTV-positive deer samples we detected originated from the counties with the highest prevalence in the aforementioned study [20]. Although no positive cases of Bluetongue virus (BTV) have been reported in our country since 2015, our findings indicate that the virus may continue to circulate asymptomatically within wild animal populations. This silent circulation poses a latent risk for the re-emergence of outbreaks. This premise is further substantiated by a study demonstrating that antibodies can be detected in deer populations even years after an initial outbreak, suggesting a potential long-term reservoir that could facilitate the reintroduction of the virus into domestic livestock populations [21].

It is important to emphasize that the sampling was carried out sporadically according to the sampling opportunity. Hence, the serological results obtained during the study are suitable for descriptive analyses only, and we cannot estimate the territorial distribution or realistic seroprevalence of the tested diseases.

Furthermore, antibody levels against the tested pathogens were not determined, since in this study, we primarily focused on the presence of data to obtain information about the serological presence of the tested viruses among wild ungulates in Hungary.

However, the seropositivity data gives a strong indication of the presence and circulation of these pathogens in the recent past or the present. The persistence mechanisms of antibodies in wild ungulates against BTV are unknown; therefore, the assessment of whether this is an unrecognized spread or whether we measured seroreactivity remaining from previous BTV epidemics is unclear [22]. Similar observations are available related to anti-flavivirus antibodies, which may persist for years. To rule out the bias of long-lasting immunity and to explore the dynamics of flavivirus spread in a given area, it is worthwhile to monitor the wild ungulate population of different ages as well [15].

Hungary’s livestock is constantly threatened by Bluetongue infection, as there is a continuous movement of animals between infected EU countries and Hungary. Cattle, which are often transported between infected and disease-free countries, show very mild or no clinical signs of the infection; however, in sheep, Bluetongue virus can be associated with much more severe symptoms [6]. From the potentially infected cattle, the virus can be transported not only to other susceptible livestock species but also to wild ruminants. So, a monitoring program that includes wild animals can indicate the presence of the virus in certain territories, and wild animals can even act like reservoir hosts of the virus [23]

The EHDV is an emerging threat in Europe with a currently ongoing spread from the Iberian Peninsula and Italy towards other parts of Europe. Since the transmitting vectors are widely present, it is likely that the virus will emerge in additional countries. In both cases (BTV and EHDV), monitoring of Culicoides vectors may serve as an efficient tool to assess and discover their presence within a country [24]. However, surveillance programs for arthropod vectors are challenging in terms of logistical resources and the processing of samples. Therefore, we believe that the procedure we propose could be a real and useful alternative to complement existing programs. Its cost-effectiveness is also noteworthy if there is nationally coordinated wildlife management activity, as it can then be implemented in conjunction with it.

## 5. Conclusions

Based on our results, we can conclude that anti-WNV and anti-BTV antibodies are present in the Hungarian fallow deer and red deer population. Despite nationwide vaccination regulation against BTV, our results highlight the silent circulation of the virus among wild ungulates, representing a constant threat to domestic ungulates.

Although the duration of the immune response against the examined viruses is unknown, the appearance of seropositivity in all study periods provides a strong basis for the conclusion that the tested pathogens are stably present within the Hungarian wild ungulate population, thus confirming the potential role of this group of animals in the monitoring system.

Our results provide a starting point for reforming the national monitoring program for the investigated pathogens, including previously neglected but potential reservoir and vector species groups in the system.

## Figures and Tables

**Table 1:** Results of ELISA and VNTs ^1^.

		European Fallow Deer *(Dama dama)*							Red Deer *(Cervus elaphus)*						
County	Locality	N.o. Animals Tested	EHDV ELISA	WNV ELISA	WNV neutr.	BTV ELISA	Seroprevalence WNV % ^2^	Seroprevalence BTV %	N.o. Animals Tested	EHDV ELISA	WNV ELISA	WNV neutr.	BTV ELISA	Seroprevalence WNV % ^2^	Seroprevalence BTV %
Bács-Kiskun	Kelebia	32	0	5	5	1	15.6	3.1	0	0	0	0	0	0	0
Baranya	Mecseknádasd	3	0	0	0	0	0	0	0	0	0	0	0	0	0
	Mészkemence	15	0	1	1	2	6.7	13.3	5	0	0	0	0	0	0
	Szentegát	1	0	1	1	0	100	0	3	0	0	0	0	0	0
Csongrád-Csanád	Hódmezővásárhely	1	0	1	1	0	100	0	0	0	0	0	0	0	0
Hajdú-Bihar	Guth	45	0	4	1	0	2.2	0	0	0	0	0	0	0	0
Nógrád	Kazár	1	0	0	0	0	0	0	0	0	0	0	0	0	0
	Pásztó	1	0	0	0	0	0	0	0	0	0	0	0	0	0
	Vizslás	1	0	0	0	0	0	0	0	0	0	0	0	0	0
Pest	Pusztavacs	1	0	0	0	0	0	0	0	0	0	0	0	0	0
Somogy	Barcs	1	0	1	0	0	0	0	0	0	0	0	0	0	0
	Csokonyavisonta	28	0	24	18	0	64.3	0	11	0	8	6	0	54.5	0
	Homokszentgyörgy	6	0	5	4	0	66.7	0	0	0	0	0	0	0	0
	Törökkoppány	31	0	13	9	2	29.0	6.5	0	0	0	0	0	0	0
Tolna	Belecska	23	0	2	2	1	8.7	4.3	0	0	0	0	0	0	0
	Gemenc	1	0	0	0	0	0	0	0	0	0	0	0	0	0
	Gyönk	21	0	6	6	0	28.6	0	2	0	1	1	0	50.0	0
	Kisszékely	7	0	3	2	0	28.6	0	0	0	0	0	0	0	0
	Kocsola	33	0	6	5	1	15.2	3	0	0	0	0	0	0	0
	Nagykónyi	9	0	6	3	0	33.3	0	0	0	0	0	0	0	0
	Szakcs	7	0	4	3	0	42.9	0	0	0	0	0	0	0	0
	Tamási	42	0	7	7	1	16.7	2.4	0	0	0	0	0	0	0
	Tolnanémedi	8	0	5	3	0	37.5	0	1	0	0	0	0	0	0
Sum		318	0	94	71	8	22.3	2.5	22	0	9	7	0	31.8	0

^1^ Samples from roe deer were excluded from the table. ^2^ Seroprevalence is counted from WNV-positive VNT data.

**Table 2:** Distribution of samples and results by sampling periods.

	European Fallow Deer		Red Deer		Roe Deer	
Sampling Period	N.o. Individuals	N.o. Positives	N.o. Individuals	N.o. Positives	N.o. Individuals	N.o. Positives
2020/2021	125	20 (WNV)	0	0	0	0
		4 (BTV)				
2021/2022	74	17 (WNV)	19	4 (WNV)	1	0
		1 (BTV)				
2022/2023	120	25 (WNV)	3	2 (WNV)	0	0
		3 (BTV)				

## Data Availability

The original contributions presented in this study are included in the article/Supplementary Materials. Further inquiries can be directed to the corresponding author(s).

## Supplementary Materials

The following supporting information can be downloaded at https://www.mdpi.com/article/10.3390/ani15131948/s1, Table S1: Metadata of sampled animals.

## Abbreviations

The following abbreviations are used in this manuscript:

BTV	Bluetongue virus
WNV	West Nile virus
EHDV	Epizootic hemorrhagic disease virus
TBEV	Tick-borne encephalitis virus
JEV	Japanese encephalitis virus
USUV	Usutu virus
ZIKAV	Zika virus
DENV	Dengue virus
ELISA	Enzyme-linked immunosorbent assay
pr-E	Envelope protein
VNT	Birus neutralization test
BSL-4	Biosafety level 4
IF	Immunofluorescence
