# Supplementary material for: Cervids as a Promising Pillar of an Integrated Surveillance System for Emerging Infectious Diseases in Hungary: A Pilot Study
Source: Animals (Basel). 2025 Jul 2;15(13):1948. doi: 10.3390/ani15131948 (PMC12248854; doi:10.3390/ani15131948)
Supplement: Supplementary file 1 [file animals-15-01948-s001.zip › animals-3557237-supplementary.pdf]

Supplementary Table1. Detailed metadata of collected samples

ID	Short ID	Species	Sex	Date	County	Geo location	WNV ELISA	WNV neutralization	BTV ELISA	EHDV ELISA
53075	GU/2	*Dama dama*	doe	2020/21	Hajdú-Bihar	Guth	0	0	0	0
300427	SZA/3	*Dama dama*	doe	2020/21	Tolna	Szakcs	1	1	0	0
309236	MÉ/1	*Dama dama*	doe	2020/21	Baranya	Mészkemence	0	0	0	0
300421	SZA/1	*Dama dama*	doe	2020/21	Tolna	Szakcs	1	1	0	0
294956	KIS/7	*Dama dama*	doe	2020/21	Tolna	Kisszékely	1	1	0	0
309234	MÉ/3	*Dama dama*	doe	2020/21	Baranya	Mészkemence	0	0	1	0
Z7072	TA/5	*Dama dama*	doe	2020/21	Tolna	Tamási	0	0	0	0
30235	NA/7	*Dama dama*	doe	2020/21	Tolna	Nagykónyi	1	0	0	0
52966	GU/3	*Dama dama*	doe	2020/21	Hajdú-Bihar	Guth	0	0	0	0
30264	NA/9	*Dama dama*	doe	2020/21	Tolna	Nagykónyi	0	0	0	0
Z6965	TA/16	*Dama dama*	doe	2020/21	Tolna	Tamási	1	1	0	0
314717	MÉ/8	*Dama dama*	doe	2020/21	Baranya	Mészkemence	0	0	0	0
314701	MÉ/7	*Dama dama*	doe	2020/21	Baranya	Mészkemence	0	0	0	0
314704	MÉ/3	*Dama dama*	doe	2020/21	Baranya	Mészkemence	0	0	0	0
46270	KE/3	*Dama dama*	doe	2020/21	Bács-Kiskun	Kelebia	0	0	0	0
294949	KIS/4	*Dama dama*	buck	2020/21	Tolna	Kisszékely	0	0	0	0
294948	KIS/3	*Dama dama*	buck	2020/21	Tolna	Kisszékely	0	0	0	0
307493	KIS/1 A	*Dama dama*	buck	2020/21	Tolna	Kisszékely	1	0	0	0
295318	KIS/1 B	*Dama dama*	buck	2020/21	Tolna	Kisszékely	0	0	0	0
295317	KIS/2	*Dama dama*	buck	2020/21	Tolna	Kisszékely	0	0	0	0
Z7011	TA/1	*Dama dama*	buck	2020/21	Tolna	Tamási	1	1	0	0
420936	HO/1	*Dama dama*	buck	2020/21	Somogy	Homokszentgyörgy	1	1	0	0
30167	NA/5	*Dama dama*	buck	2020/21	Tolna	Nagykónyi	1	1	0	0
421710	TÖ/7	*Dama dama*	buck	2020/21	Somogy	Törökkoppány	1	0	0	0
421712	TÖ/6	*Dama dama*	buck	2020/21	Somogy	Törökkoppány	1	1	0	0
300423	SZA/22	*Dama dama*	buck	2020/21	Tolna	Szakcs	1	1	0	0

300422	SZA/2	*Dama dama*	buck	2020/21	Tolna	Szakcs	0	0	0	0
428922	BR/1	*Dama dama*	buck	2020/21	Somogy	Barcs	1	0	0	0
30017	NA/3	*Dama dama*	buck	2020/21	Tolna	Nagykónyi	0	0	0	0
30114	NA/4	*Dama dama*	buck	2020/21	Tolna	Nagykónyi	1	0	0	0
421688	TÖ/1	*Dama dama*	buck	2020/21	Somogy	Törökkoppány	1	0	0	0
Z7029	TA/3	*Dama dama*	buck	2020/21	Tolna	Tamási	0	0	1	0
295345	KIS/5	*Dama dama*	buck	2020/21	Tolna	Kisszékely	1	1	0	0
421659	TÖ/12	*Dama dama*	buck	2020/21	Somogy	Törökkoppány	1	0	0	0
421680	TÖ/3	*Dama dama*	buck	2020/21	Somogy	Törökkoppány	1	1	0	0
421713	TÖ/10	*Dama dama*	buck	2020/21	Somogy	Törökkoppány	1	1	1	0
Z7028	TA/2	*Dama dama*	doe	2020/21	Tolna	Tamási	1	1	0	0
464365	KE/16	*Dama dama*	doe	2020/21	Bács-Kiskun	Kelebia	0	0	0	0
464368	KE/15	*Dama dama*	doe	2020/21	Bács-Kiskun	Kelebia	0	0	0	0
464367	KE/14	*Dama dama*	doe	2020/21	Bács-Kiskun	Kelebia	0	0	0	0
464371	KE/17	*Dama dama*	doe	2020/21	Bács-Kiskun	Kelebia	0	0	0	0
464369	KE/18	*Dama dama*	doe	2020/21	Bács-Kiskun	Kelebia	0	0	0	0
464364	KE/19	*Dama dama*	doe	2020/21	Bács-Kiskun	Kelebia	0	0	0	0
421458	TÖ/11	*Dama dama*	buck	2020/21	Somogy	Törökkoppány	1	1	0	0
421711	TÖ/5	*Dama dama*	buck	2020/21	Somogy	Törökkoppány	1	0	0	0
421714	TÖ/9	*Dama dama*	buck	2020/21	Somogy	Törökkoppány	1	1	1	0
443229	TO/7	*Dama dama*	buck	2020/21	Tolna	Tolnanémedi	1	1	0	0
341076	PU/1	*Dama dama*	buck	2020/21	Pest	Pusztavacs	0	0	0	0
283472	PÁ/1	*Dama dama*	buck	2020/21	Nógrád	Pásztó	0	0	0	0
443227	TO/6	*Dama dama*	buck	2020/21	Tolna	Tolnanémedi	0	0	0	0
32118	TA/16	*Dama dama*	doe	2020/21	Tolna	Tamási	0	0	0	0
32116	TA/14	*Dama dama*	doe	2020/21	Tolna	Tamási	1	1	0	0
304818	TÖ/11	*Dama dama*	doe	2020/21	Somogy	Törökkoppány	0	0	0	0
Z32121	TA/19	*Dama dama*	doe	2020/21	Tolna	Tamási	0	0	0	0
304820	TÖ/12	*Dama dama*	doe	2020/21	Somogy	Törökkoppány	0	0	0	0
304821	TÖ/13	*Dama dama*	doe	2020/21	Somogy	Törökkoppány	0	0	0	0

Z32122	TA/20	*Dama dama*	doe	2020/21	Tolna	Tamási	0	0	0	0
Z32119	TA/17	*Dama dama*	doe	2020/21	Tolna	Tamási	0	0	0	0
Z32123	TA/21	*Dama dama*	doe	2020/21	Tolna	Tamási	0	0	0	0
304827	TÖ/16	*Dama dama*	doe	2020/21	Somogy	Törökkoppány	0	0	0	0
Z32124	TA/22	*Dama dama*	doe	2020/21	Tolna	Tamási	0	0	0	0
Z32115	TA/15	*Dama dama*	doe	2020/21	Tolna	Tamási	0	0	0	0
Z32120	TA/18	*Dama dama*	doe	2020	Tolna	Tamási	0	0	0	0
299190	K/29	*Dama dama*	doe	2020	Tolna	Kocsola	0	0	0	0
299189	K/28	*Dama dama*	doe	2020	Tolna	Kocsola	0	0	0	0
299196	K/35	*Dama dama*	doe	2020	Tolna	Kocsola	1	1	0	0
299197	K/36	*Dama dama*	doe	2020	Tolna	Kocsola	0	0	0	0
299193	K/32	*Dama dama*	doe	2020	Tolna	Kocsola	0	0	0	0
414597	ME/3	*Dama dama*	doe	2020	Baranya	Mecseknádasd	0	0	0	0
299186	K/25	*Dama dama*	doe	2020	Tolna	Kocsola	1	1	0	0
414520	ME/1	*Dama dama*	doe	2020	Baranya	Mecseknádasd	0	0	0	0
299187	K/26	*Dama dama*	doe	2020	Tolna	Kocsola	0	0	0	0
299192	K/31	*Dama dama*	doe	2020	Tolna	Kocsola	0	0	0	0
299188	K/27	*Dama dama*	doe	2020	Tolna	Kocsola	0	0	0	0
299194	K/33	*Dama dama*	doe	2020	Tolna	Kocsola	1	0	0	0
299191	K/30	*Dama dama*	doe	2020	Tolna	Kocsola	0	0	0	0
414599	ME/2	*Dama dama*	doe	2020	Baranya	Mecseknádasd	0	0	0	0
299195	K/34	*Dama dama*	doe	2020	Tolna	Kocsola	0	0	0	0
443226	TO/5	*Dama dama*	buck	2020	Tolna	Tolnanémedi	1	0	0	0
297345	N/7	*Dama dama*	buck	2020	Tolna	Nagykónyi	1	1	0	0
283290	V/1	*Dama dama*	buck	2020	Nógrád	Vizslás	0	0	0	0
287197	KA/1	*Dama dama*	buck	2020	Nógrád	Kazár	0	0	0	0
297436	N/8	*Dama dama*	buck	2020	Tolna	Nagykónyi	1	1	0	0
295544	SZ/6	*Dama dama*	buck	2020	Tolna	Szakcs	1	0	0	0
253256	GY/15	*Dama dama*	doe	2020	Tolna	Gyönk	0	0	0	0
203266	GY/13	*Dama dama*	doe	2020	Tolna	Gyönk	0	0	0	0

203278	GY/11	*Dama dama*	doe	2020	Tolna	Gyönk	1	1	0	0
203271	GY/10	*Dama dama*	doe	2020	Tolna	Gyönk	1	1	0	0
203262	GY/12	*Dama dama*	doe	2020	Tolna	Gyönk	0	0	0	0
203279	GY/14	*Dama dama*	doe	2020	Tolna	Gyönk	0	0	0	0
486660	GYÖ/5	*Dama dama*	doe	2021	Tolna	Gyönk	0	0	0	0
486757	GYÖ/10	*Dama dama*	doe	2021	Tolna	Gyönk	0	0	0	0
456758	GYÖ/16	*Dama dama*	doe	2021	Tolna	Gyönk	0	0	0	0
486755	GYÖ/15	*Dama dama*	doe	2021	Tolna	Gyönk	0	0	0	0
Z7076	TA/10	*Dama dama*	doe	2021	Tolna	Tamási	0	0	0	0
53655	GU/1	*Dama dama*	doe	2021	Hajdú-Bihar	Guth	0	0	0	0
483215	TO/4	*Dama dama*	buck	2021	Tolna	Tolnanémedi	0	0	0	0
30196	NA/6	*Dama dama*	buck	2021	Tolna	Nagykónyi	0	0	0	0
426709	Ke/1	*Dama dama*	buck	2021	Bács-Kiskun	Kelebia	0	0	0	0
300428	SZA/4	*Dama dama*	buck	2021	Tolna	Szakcs	0	0	0	0
486760	GYÖ/17	*Dama dama*	buck	2021	Tolna	Gyönk	0	0	0	0
483321	TO/5	*Dama dama*	buck	2021	Tolna	Tolnanémedi	1	0	0	0
483323	TO/6	*Dama dama*	buck	2021	Tolna	Tolnanémedi	1	1	0	0
30120	NA/8	*Dama dama*	buck	2021	Tolna	Nagykónyi	1	0	0	0
483549	KO/5	*Dama dama*	doe	2021	Tolna	Kocsola	0	0	0	0
421870	TÖ/3	*Dama dama*	doe	2021	Somogy	Törökkoppány	0	0	0	0
421856	TÖ/8	*Dama dama*	doe	2021	Somogy	Törökkoppány	0	0	0	0
421861	TÖ/6	*Dama dama*	doe	2021	Somogy	Törökkoppány	0	0	0	0
Z6964	TA/15	*Dama dama*	doe	2021	Tolna	Tamási	0	0	0	0
421995	TÖ/12	*Dama dama*	doe	2021	Somogy	Törökkoppány	1	1	0	0
226005	GU/16	*Dama dama*	doe	2021	Hajdú-Bihar	Guth	0	0	0	0
226035	GU/18	*Dama dama*	doe	2021	Hajdú-Bihar	Guth	0	0	0	0
225989	GU/13	*Dama dama*	doe	2021	Hajdú-Bihar	Guth	0	0	0	0
Z7078	TA/9	*Dama dama*	doe	2021	Tolna	Tamási	0	0	0	0
Z7133	TA/7	*Dama dama*	doe	2021	Tolna	Tamási	0	0	0	0
Z6962	TA/12	*Dama dama*	doe	2021	Tolna	Tamási	0	0	0	0

Z6963	TA/13	*Dama dama*	doe	2021	Tolna	Tamási	0	0	0	0
Z7134	TA/11	*Dama dama*	doe	2021	Tolna	Tamási	0	0	0	0
Z7075	TA/6	*Dama dama*	doe	2021	Tolna	Tamási	0	0	0	0
Z7077	TA/8	*Dama dama*	doe	2021	Tolna	Tamási	0	0	0	0
53674	GU/8	*Dama dama*	doe	2021	Hajdú-Bihar	Guth	0	0	0	0
53681	GU/7	*Dama dama*	doe	2021	Hajdú-Bihar	Guth	0	0	0	0
53658	GU/4	*Dama dama*	doe	2021	Hajdú-Bihar	Guth	0	0	0	0
53651	GU/5	*Dama dama*	doe	2021	Hajdú-Bihar	Guth	0	0	0	0
199579	GE/5	*Dama dama*	doe	2021/22	Tolna	Gemenc	0	0	0	0
276296	CSO/3	*Cervus elaphus*	juvenile hind	2021/22	Somogy	Csokonyavisonta	0	0	0	0
276297	CSO/4	*Cervus elaphus*	juvenile hind	2021/22	Somogy	Csokonyavisonta	0	0	0	0
14649	MÉ/4	*Cervus elaphus*	juvenile hind	2021/22	Baranya	Mészkemence	0	0	0	0
14648	MÉ/3	*Cervus elaphus*	juvenile hind	2021/22	Baranya	Mészkemence	0	0	0	0
439151	KE/9	*Dama dama*	doe	2021/22	Bács-Kiskun	Kelebia	0	0	0	0
439150	KE/7	*Dama dama*	doe	2021/22	Bács-Kiskun	Kelebia	0	0	0	0
439155	KE/8	*Dama dama*	doe	2021/22	Bács-Kiskun	Kelebia	0	0	0	0
295283	MÉ/1	*Dama dama*	doe	2021/22	Baranya	Mészkemence	0	0	1	0
439154	KE/10	*Dama dama*	doe	2021/22	Bács-Kiskun	Kelebia	0	0	0	0
439152	KE/12	*Dama dama*	doe	2021/22	Bács-Kiskun	Kelebia	0	0	0	0
200250	KO/13	*Dama dama*	doe	2021/22	Tolna	Kocsola	0	0	0	0
200256	KO/8	*Dama dama*	doe	2021/22	Tolna	Kocsola	0	0	0	0
200274	KO/9	*Dama dama*	doe	2021/22	Tolna	Kocsola	0	0	0	0
51746	GU/4	*Dama dama*	doe	2021/22	Hajdú-Bihar	Guth	0	0	0	0
14692	MÉ/1	*Cervus elaphus*	juvenile hind	2021/22	Baranya	Mészkemence	0	0	0	0
14691	MÉ/2	*Cervus elaphus*	juvenile hind	2021/22	Baranya	Mészkemence	0	0	0	0
292935	TÖ/2	*Dama dama*	juvenile doe	2021/22	Somogy	Törökkoppány	0	0	0	0
15707	SZE/1	*Cervus elaphus*	juvenile hind	2021/22	Baranya	Szentegát	0	0	0	0
51736	GU/9	*Dama dama*	doe	2021/22	Hajdú-Bihar	Guth	0	0	0	0
51740	GU/10	*Dama dama*	doe	2021/22	Hajdú-Bihar	Guth	0	0	0	0
51739	GU/8	*Dama dama*	doe	2021/22	Hajdú-Bihar	Guth	0	0	0	0

188427	BE/6	*Dama dama*	doe	2021/22	Tolna	Belecska	0	0	0	0
188402	BE/1	*Dama dama*	doe	2021/22	Tolna	Belecska	0	0	0	0
188425	BE/4	*Dama dama*	doe	2021/22	Tolna	Belecska	1	1	0	0
188418	BE/3	*Dama dama*	doe	2021/22	Tolna	Belecska	0	0	0	0
188426	BE/5	*Dama dama*	doe	2021/22	Tolna	Belecska	1	1	0	0
188404	BE/2	*Dama dama*	doe	2021/22	Tolna	Belecska	0	0	0	0
301722	CSO/9	*Dama dama*	doe	2021/22	Somogy	Csokonyavisonta	1	1	0	0
301797	CSO/7	*Dama dama*	doe	2021/22	Somogy	Csokonyavisonta	0	0	0	0
301724	CSO/13	*Dama dama*	doe	2021/22	Somogy	Csokonyavisonta	1	1	0	0
209896	SZA/1	*Dama dama*	doe	2021/22	Tolna	Szakcs	0	0	0	0
186907	TO/3	*Dama dama*	doe	2021/22	Tolna	Tolnanémedi	1	1	0	0
245928	HO/1	*Dama dama*	buck	2021/22	Csongrád-Csanád	Hódmezővásárhely	1	1	0	0
189853	TO/1	*Capreolus capreolus*	buck	2021/22	Tolna	Tolnanémedi	0	0	0	0
189857	TO/2	*Dama dama*	doe	2021/22	Tolna	Tolnanémedi	0	0	0	0
335469	TÖ/3	*Dama dama*	doe	2021/22	Somogy	Törökkoppány	0	0	0	0
335456	TÖ/1	*Dama dama*	doe	2021/22	Somogy	Törökkoppány	0	0	0	0
439039	KE/4	*Dama dama*	doe	2021/22	Bács-Kiskun	Kelebia	0	0	0	0
439060	KE/3	*Dama dama*	doe	2021/22	Bács-Kiskun	Kelebia	0	0	0	0
439037	KE/1	*Dama dama*	doe	2021/22	Bács-Kiskun	Kelebia	0	0	0	0
439042	KE/5	*Dama dama*	doe	2021/22	Bács-Kiskun	Kelebia	1	1	0	0
439038	KE/2	*Dama dama*	doe	2021/22	Bács-Kiskun	Kelebia	1	1	0	0
52268	GU/11	*Dama dama*	doe	2021/22	Hajdú-Bihar	Guth	0	0	0	0
52214	GU/20	*Dama dama*	doe	2021/22	Hajdú-Bihar	Guth	0	0	0	0
52267	GU/13	*Dama dama*	doe	2021/22	Hajdú-Bihar	Guth	0	0	0	0
334239	HO/16	*Dama dama*	doe	2021/22	Somogy	Homokszentgyörgy	0	0	0	0
334236	HO/11	*Dama dama*	doe	2021/22	Somogy	Homokszentgyörgy	1	0	0	0
334238	HO/15	*Dama dama*	doe	2021/22	Somogy	Homokszentgyörgy	1	1	0	0
334234	HO/18	*Dama dama*	doe	2021/22	Somogy	Homokszentgyörgy	1	1	0	0
15710	SZE/2	*Cervus elaphus*	juvenile hind	2021/22	Baranya	Szentegát	0	0	0	0
276273	CSO/2	*Cervus elaphus*	juvenile hind	2021/22	Somogy	Csokonyavisonta	0	0	0	0

276272	CSO/1	*Cervus elaphus*	juvenile hind	2021/22	Somogy	Csokonyavisonta	1	1	0	0
292932	TÖ/1	*Dama dama*	juvenile doe	2021/22	Somogy	Törökkoppány	0	0	0	0
200256	KO/2	*Dama dama*	doe	2021/22	Tolna	Kocsola	0	0	0	0
200264	KO/4	*Dama dama*	doe	2021/22	Tolna	Kocsola	1	1	0	0
200262	KO/6	*Dama dama*	doe	2021/22	Tolna	Kocsola	0	0	0	0
200257	KO/7	*Dama dama*	doe	2021/22	Tolna	Kocsola	0	0	0	0
200261	KO/3	*Dama dama*	doe	2021/22	Tolna	Kocsola	1	1	0	0
188434	BE/12	*Dama dama*	doe	2021/22	Tolna	Belecska	0	0	0	0
188432	BE/10	*Dama dama*	doe	2021/22	Tolna	Belecska	0	0	0	0
14731	MÉ/5	*Cervus elaphus*	juvenile hind	2021/22	Baranya	Mészkemence	0	0	0	0
15709	SZE/.3	*Cervus elaphus*	juvenile hind	2021/22	Baranya	Szentegát	0	0	0	0
39682	TO/1	*Cervus elaphus*	juvenile hind	2021/22	Tolna	Tolnanémedi	0	0	0	0
276191	CSO/5	*Cervus elaphus*	juvenile hind	2021/22	Somogy	Csokonyavisonta	1	1	0	0
334213	HO/2	*Dama dama*	doe	2021/22	Somogy	Homokszentgyörgy	1	1	0	0
300412	CSO/3	*Dama dama*	buck	2021/22	Somogy	Csokonyavisonta	1	1	0	0
293185	G/9	*Dama dama*	buck	2021/22	Somogy	Törökkoppány	1	1	0	0
293184	TÖ/3	*Dama dama*	buck	2021/22	Somogy	Törökkoppány	0	0	0	0
14773	MÉ/2	*Dama dama*	buck	2021/22	Baranya	Mészkemence	1	1	0	0
220675	KE/3	*Dama dama*	buck	2021/22	Bács-Kiskun	Kelebia	0	0	0	0
300612	CSO/1	*Dama dama*	buck	2021/22	Somogy	Csokonyavisonta	0	0	0	0
300512	CSO/4	*Dama dama*	buck	2021/22	Somogy	Csokonyavisonta	1	0	0	0
14767	MÉ/1	*Dama dama*	buck	2021/22	Baranya	Mészkemence	0	0	0	0
14768	MÉ/3	*Dama dama*	buck	2021/22	Baranya	Mészkemence	0	0	0	0
220674	KE/4	*Dama dama*	buck	2021/22	Bács-Kiskun	Kelebia	0	0	0	0
220672	KE/1	*Dama dama*	buck	2021/22	Bács-Kiskun	Kelebia	1	1	0	0
220673	KE/5	*Dama dama*	buck	2021/22	Bács-Kiskun	Kelebia	0	0	0	0
220678	KE/6	*Dama dama*	buck	2021/22	Bács-Kiskun	Kelebia	0	0	0	0
220680	KE/2	*Dama dama*	buck	2021/22	Bács-Kiskun	Kelebia	1	1	0	0
220677	KE/7	*Dama dama*	buck	2021/22	Bács-Kiskun	Kelebia	0	0	0	0
444906	GU/6	*Dama dama*	buck	2021/22	Hajdú-Bihar	Guth	1	0	0	0

444953	GU/1	*Dama dama*	buck	2021/22	Hajdú-Bihar	Guth	0	0	0	0
444877	GU/8	*Dama dama*	buck	2021/22	Hajdú-Bihar	Guth	0	0	0	0
444955	GU/7	*Dama dama*	buck	2021/22	Hajdú-Bihar	Guth	0	0	0	0
444901	GU/4	*Dama dama*	buck	2021/22	Hajdú-Bihar	Guth	1	0	0	0
444884	GU/5	*Dama dama*	buck	2021/22	Hajdú-Bihar	Guth	1	1	0	0
444907	GU/2	*Dama dama*	buck	2021/22	Hajdú-Bihar	Guth	0	0	0	0
444945	GU/3	*Dama dama*	buck	2021/22	Hajdú-Bihar	Guth	0	0	0	0
300507	G/7	*Cervus elaphus*	stag	2021/22	Somogy	Csokonyavisonta	1	1	0	0
300508	G/5	*Cervus elaphus*	stag	2021/22	Somogy	Csokonyavisonta	1	1	0	0
300604	G/6	*Cervus elaphus*	stag	2021/22	Somogy	Csokonyavisonta	1	0	0	0
300406	G/4	*Cervus elaphus*	stag	2021/22	Somogy	Csokonyavisonta	1	0	0	0
300510	G/8	*Cervus elaphus*	stag	2021/22	Somogy	Csokonyavisonta	1	1	0	0
447249	GU/26	*Dama dama*	doe	2022/2023	Hajdú-Bihar	Guth	0	0	0	0
447242	GU/25	*Dama dama*	doe	2022/2023	Hajdú-Bihar	Guth	0	0	0	0
447258	GU/28	*Dama dama*	doe	2022/2023	Hajdú-Bihar	Guth	0	0	0	0
447245	GU/24	*Dama dama*	doe	2022/2023	Hajdú-Bihar	Guth	0	0	0	0
447287	GU/21	*Dama dama*	doe	2022/2023	Hajdú-Bihar	Guth	0	0	0	0
445667	GU/20	*Dama dama*	doe	2022/2023	Hajdú-Bihar	Guth	0	0	0	0
447703	GU/27	*Dama dama*	doe	2022/2023	Hajdú-Bihar	Guth	0	0	0	0
447306	GU/19	*Dama dama*	doe	2022/2023	Hajdú-Bihar	Guth	0	0	0	0
447313	GU/22	*Dama dama*	doe	2022/2023	Hajdú-Bihar	Guth	0	0	0	0
447304	GU/23	*Dama dama*	doe	2022/2023	Hajdú-Bihar	Guth	0	0	0	0
300380	CSO/21	*Dama dama*	doe	2022/2023	Somogy	Csokonyavisonta	1	0	0	0
300391	CSO/20	*Dama dama*	doe	2022/2023	Somogy	Csokonyavisonta	1	0	0	0
300396	CSO/24	*Dama dama*	doe	2022/2023	Somogy	Csokonyavisonta	0	0	0	0
300574	CSO/23	*Dama dama*	doe	2022/2023	Somogy	Csokonyavisonta	1	1	0	0
300385	CSO/22	*Cervus elaphus*	hind	2022/2023	Somogy	Csokonyavisonta	1	1	0	0
300361	CSO/28	*Dama dama*	doe	2022/2023	Somogy	Csokonyavisonta	1	1	0	0
300362	CSO/27	*Dama dama*	doe	2022/2023	Somogy	Csokonyavisonta	1	0	0	0
300373	CSO/26	*Dama dama*	doe	2022/2023	Somogy	Csokonyavisonta	1	0	0	0

300387	CSO/25	*Dama dama*	doe	2022/2023	Somogy	Csokonyavisonta	0	0	0	0
447296	GU/12	*Dama dama*	doe	2022/2023	Hajdú-Bihar	Guth	0	0	0	0
445610	GU/10	*Dama dama*	doe	2022/2023	Hajdú-Bihar	Guth	0	0	0	0
447254	GU/9	*Dama dama*	doe	2022/2023	Hajdú-Bihar	Guth	0	0	0	0
447252	GU/14	*Dama dama*	doe	2022/2023	Hajdú-Bihar	Guth	0	0	0	0
445640	GU/13	*Dama dama*	doe	2022/2023	Hajdú-Bihar	Guth	0	0	0	0
447203	GU/16	*Dama dama*	doe	2022/2023	Hajdú-Bihar	Guth	0	0	0	0
445637	GU/17	*Dama dama*	doe	2022/2023	Hajdú-Bihar	Guth	0	0	0	0
447209	GU/15	*Dama dama*	doe	2022/2023	Hajdú-Bihar	Guth	0	0	0	0
445612	GU/18	*Dama dama*	doe	2022/2023	Hajdú-Bihar	Guth	0	0	0	0
445635	GU/11	*Dama dama*	doe	2022/2023	Hajdú-Bihar	Guth	1	0	0	0
300452	CSO/16	*Dama dama*	doe	2022/2023	Somogy	Csokonyavisonta	1	1	0	0
300688	CSO/15	*Dama dama*	doe	2022/2023	Somogy	Csokonyavisonta	1	1	0	0
300456	CSO/19	*Dama dama*	doe	2022/2023	Somogy	Csokonyavisonta	1	1	0	0
300686	CSO/13	*Dama dama*	doe	2022/2023	Somogy	Csokonyavisonta	0	0	0	0
300684	CSO/14	*Dama dama*	doe	2022/2023	Somogy	Csokonyavisonta	1	1	0	0
300453	CSO/17	*Dama dama*	doe	2022/2023	Somogy	Csokonyavisonta	1	1	0	0
300524	CSO/9	*Dama dama*	doe	2022/2023	Somogy	Csokonyavisonta	1	1	0	0
300527	CSO/11	*Dama dama*	doe	2022/2023	Somogy	Csokonyavisonta	1	1	0	0
300523	CSO/8	*Dama dama*	doe	2022/2023	Somogy	Csokonyavisonta	1	1	0	0
300528	CSO/12	*Dama dama*	doe	2022/2023	Somogy	Csokonyavisonta	1	1	0	0
300516	CSO/5	*Dama dama*	doe	2022/2023	Somogy	Csokonyavisonta	1	1	0	0
300518	CSO/6	*Dama dama*	doe	2022/2023	Somogy	Csokonyavisonta	1	1	0	0
300525	CSO/10	*Dama dama*	doe	2022/2023	Somogy	Csokonyavisonta	1	1	0	0
300522	CSO/7	*Dama dama*	doe	2022/2023	Somogy	Csokonyavisonta	1	1	0	0
397630	BE/8	*Dama dama*	doe	2022/2023	Tolna	Belecska	0	0	0	0
397629	BE/7	*Dama dama*	doe	2022/2023	Tolna	Belecska	0	0	0	0
397632	BE/10	*Dama dama*	doe	2022/2023	Tolna	Belecska	0	0	0	0
397623	BE/1	*Dama dama*	doe	2022/2023	Tolna	Belecska	0	0	0	0
397628	BE/6	*Dama dama*	doe	2022/2023	Tolna	Belecska	0	0	0	0

397626	BE/4	*Dama dama*	doe	2022/2023	Tolna	Belecska	0	0	0	0
397633	BE/11	*Dama dama*	doe	2022/2023	Tolna	Belecska	0	0	0	0
397631	BE/9	*Dama dama*	doe	2022/2023	Tolna	Belecska	0	0	0	0
397624	BE/2	*Dama dama*	doe	2022/2023	Tolna	Belecska	0	0	0	0
472464	BE/15	*Dama dama*	doe	2022/2023	Tolna	Belecska	0	0	0	0
472461	BE/12	*Dama dama*	doe	2022/2023	Tolna	Belecska	0	0	0	0
472465	BE/16	*Dama dama*	doe	2022/2023	Tolna	Belecska	0	0	0	0
472462	BE/13	*Dama dama*	doe	2022/2023	Tolna	Belecska	0	0	0	0
472463	BE/14	*Dama dama*	doe	2022/2023	Tolna	Belecska	0	0	0	0
472466	BE/17	*Dama dama*	doe	2022/2023	Tolna	Belecska	0	0	1	0
395042	GYÖ/4	*Dama dama*	doe	2022/2023	Tolna	Gyönk	0	0	0	0
395048	GYÖ/5	*Dama dama*	doe	2022/2023	Tolna	Gyönk	1	1	0	0
395036	GYÖ/1	*Dama dama*	doe	2022/2023	Tolna	Gyönk	1	1	0	0
395037	GYÖ/2	*Cervus elaphus*	hind	2022/2023	Tolna	Gyönk	1	1	0	0
395041	GYÖ/3	*Dama dama*	doe	2022/2023	Tolna	Gyönk	1	1	0	0
399429	KO/4	*Dama dama*	doe	2022/2023	Tolna	Kocsola	0	0	0	0
399445	KO/7	*Dama dama*	doe	2022/2023	Tolna	Kocsola	0	0	0	0
399428	KO/3	*Dama dama*	doe	2022/2023	Tolna	Kocsola	0	0	0	0
399426	KO/1	*Dama dama*	doe	2022/2023	Tolna	Kocsola	1	1	0	0
399448	KO/10	*Dama dama*	doe	2022/2023	Tolna	Kocsola	0	0	0	0
399427	KO/2	*Dama dama*	doe	2022/2023	Tolna	Kocsola	0	0	0	0
399449	KO/11	*Dama dama*	doe	2022/2023	Tolna	Kocsola	0	0	1	0
399440	KO/5	*Dama dama*	doe	2022/2023	Tolna	Kocsola	0	0	0	0
399447	KO/9	*Dama dama*	doe	2022/2023	Tolna	Kocsola	0	0	0	0
399441	KO/6	*Dama dama*	doe	2022/2023	Tolna	Kocsola	0	0	0	0
399446	KO/8	*Dama dama*	doe	2022/2023	Tolna	Kocsola	0	0	0	0
399450	KO/12	*Dama dama*	doe	2022/2023	Tolna	Kocsola	0	0	0	0
395147	GYÖ/12	*Cervus elaphus*	hind	2022/2023	Tolna	Gyönk	0	0	0	0
395110	GYÖ/7	*Dama dama*	doe	2022/2023	Tolna	Gyönk	0	0	0	0
395117	GYÖ/9	*Dama dama*	doe	2022/2023	Tolna	Gyönk	0	0	0	0

395119	GYÖ/10	*Dama dama*	doe	2022/2023	Tolna	Gyönk	1	1	0	0
395104	GYÖ/6	*Dama dama*	doe	2022/2023	Tolna	Gyönk	0	0	0	0
395111	GYÖ/8	*Dama dama*	doe	2022/2023	Tolna	Gyönk	0	0	0	0
395146	GYÖ/11	*Dama dama*	doe	2022/2023	Tolna	Gyönk	0	0	0	0
15745	SZE/4	*Dama dama*	doe	2022/2023	Baranya	Szentegát	1	1	0	0
23559	MÉ/4	*Dama dama*	doe	2022/2023	Baranya	Mészkemence	0	0	0	0
23505	MÉ/6	*Dama dama*	doe	2022/2023	Baranya	Mészkemence	0	0	0	0
23560	MÉ/5	*Dama dama*	doe	2022/2023	Baranya	Mészkemence	0	0	0	0
23577	MÉ/8	*Dama dama*	doe	2022/2023	Baranya	Mészkemence	0	0	0	0
23538	MÉ/9	*Dama dama*	doe	2022/2023	Baranya	Mészkemence	0	0	0	0
23539	MÉ/10	*Dama dama*	doe	2022/2023	Baranya	Mészkemence	0	0	0	0
293264	TÖ/9	*Dama dama*	doe	2022/2023	Somogy	Törökkoppány	0	0	0	0
293263	TÖ/8	*Dama dama*	doe	2022/2023	Somogy	Törökkoppány	1	1	0	0
293261	TÖ/6	*Dama dama*	doe	2022/2023	Somogy	Törökkoppány	0	0	0	0
293260	TÖ/5	*Dama dama*	doe	2022/2023	Somogy	Törökkoppány	0	0	0	0
293256	TÖ/1	*Dama dama*	doe	2022/2023	Somogy	Törökkoppány	0	0	0	0
293257	TÖ/2	*Dama dama*	doe	2022/2023	Somogy	Törökkoppány	0	0	0	0
293262	TÖ/7	*Dama dama*	doe	2022/2023	Somogy	Törökkoppány	0	0	0	0
293258	TÖ/3	*Dama dama*	doe	2022/2023	Somogy	Törökkoppány	1	1	0	0
220939	KE/14	*Dama dama*	doe	2022/2023	Bács-Kiskun	Kelebia	1	1	0	0
220850	KE/11	*Dama dama*	doe	2022/2023	Bács-Kiskun	Kelebia	0	0	0	0
220816	KE/8	*Dama dama*	doe	2022/2023	Bács-Kiskun	Kelebia	0	0	0	0
220819	KE/10	*Dama dama*	doe	2022/2023	Bács-Kiskun	Kelebia	0	0	0	0
220933	KE/13	*Dama dama*	doe	2022/2023	Bács-Kiskun	Kelebia	0	0	0	0
220818	KE/9	*Dama dama*	doe	2022/2023	Bács-Kiskun	Kelebia	0	0	0	0
220849	KE/12	*Dama dama*	doe	2022/2023	Bács-Kiskun	Kelebia	0	0	1	0
Z24307	TA/23	*Dama dama*	doe	2022/2023	Tolna	Tamási	1	1	0	0
Z24308	TA/24	*Dama dama*	doe	2022/2023	Tolna	Tamási	0	0	0	0
Z24304	TA/20	*Dama dama*	doe	2022/2023	Tolna	Tamási	1	1	0	0
Z24302	TA/18	*Dama dama*	doe	2022/2023	Tolna	Tamási	0	0	0	0

Z24305	TA/21	*Dama dama*	doe	2022/2023	Tolna	Tamási	0	0	0	0
Z24306	TA/22	*Dama dama*	doe	2022/2023	Tolna	Tamási	0	0	0	0
Z24303	TA/19	*Dama dama*	doe	2022/2023	Tolna	Tamási	0	0	0	0
Z24464	TA/14	*Dama dama*	doe	2022/2023	Tolna	Tamási	0	0	0	0
Z24466	TA/16	*Dama dama*	doe	2022/2023	Tolna	Tamási	0	0	0	0
Z24463	TA/13	*Dama dama*	doe	2022/2023	Tolna	Tamási	0	0	0	0
Z24585	TA/7	*Dama dama*	doe	2022/2023	Tolna	Tamási	0	0	0	0
Z24465	TA/15	*Dama dama*	doe	2022/2023	Tolna	Tamási	0	0	0	0
Z24584	TA/6	*Dama dama*	doe	2022/2023	Tolna	Tamási	0	0	0	0
Z24588	TA/10	*Dama dama*	doe	2022/2023	Tolna	Tamási	1	1	0	0
Z24468	TA/17	*Dama dama*	doe	2022/2023	Tolna	Tamási	0	0	0	0
Z24589	TA/11	*Dama dama*	doe	2022/2023	Tolna	Tamási	0	0	0	0
Z24587	TA/9	*Dama dama*	doe	2022/2023	Tolna	Tamási	0	0	0	0
Z24586	TA/8	*Dama dama*	doe	2022/2023	Tolna	Tamási	0	0	0	0
Z24462	TA/12	*Dama dama*	doe	2022/2023	Tolna	Tamási	0	0	0	0
